# *p*-wave triggered superconductivity in single-layer graphene on an electron-doped oxide superconductor

**DOI:** 10.1038/ncomms14024

**Published:** 2017-01-19

**Authors:** A. Di Bernardo, O. Millo, M. Barbone, H. Alpern, Y. Kalcheim, U. Sassi, A. K. Ott, D. De Fazio, D. Yoon, M. Amado, A. C. Ferrari, J. Linder, J. W. A. Robinson

**Affiliations:** 1 Department of Materials Science and Metallurgy, University of Cambridge, 27 Charles Babbage Road, Cambridge CB3 0FS, UK; 2 Racah Institute of Physics and the Hebrew University Center for Nanoscience and Nanotechnology, The Hebrew University of Jerusalem, Jerusalem 91904, Israel; 3 Cambridge Graphene Centre, University of Cambridge, Cambridge CB3 0FA, UK; 4 Department of Physics, Norwegian University of Science and Technology, N-7491 Trondheim, Norway

## Abstract

Electron pairing in the vast majority of superconductors follows the Bardeen–Cooper–Schrieffer theory of superconductivity, which describes the condensation of electrons into pairs with antiparallel spins in a singlet state with an *s*-wave symmetry. Unconventional superconductivity was predicted in single-layer graphene (SLG), with the electrons pairing with a *p*-wave or chiral *d*-wave symmetry, depending on the position of the Fermi energy with respect to the Dirac point. By placing SLG on an electron-doped (non-chiral) *d*-wave superconductor and performing local scanning tunnelling microscopy and spectroscopy, here we show evidence for a *p*-wave triggered superconducting density of states in SLG. The realization of unconventional superconductivity in SLG offers an exciting new route for the development of *p*-wave superconductivity using two-dimensional materials with transition temperatures above 4.2 K.

## Abstract (web summary)

Unconventional superconductivity may be triggered when graphene is deposited on a high temperature superconductor. Here, Di Bernardo *et al*. observe spectroscopic evidence for *p*-wave superconductivity in single layer graphene on an electron-doped cuprate superconductor.

Electron pairing in the vast majority of superconductors follows the Bardeen, Cooper and Schrieffer (BCS) theory of superconductivity^1^, which describes the condensation of electron pairs with opposite spins in a singlet state with an isotropic order parameter (*s*-wave symmetry). Superconductivity in a number of compounds is predicted to be unconventional in that the electrons pair in a triplet state with parallel spins and anisotropic *p*-wave symmetry, most notably in UPt_3_ (ref. 2) and Sr_2_RuO_4_ (SRO)^3^,^4^. Although the investigation of compounds that are thought to be bulk *p*-wave superconductors has been intensive over the past two decades^5^,^6^ there is still much debate over the nature of the superconductivity in these compounds. Theoretically, a *p*-wave superconducting state is highly sensitive to electron scattering which has meant that, with exception to ref. 7, all experiments to date on potential *p*-wave superconductors have focused on single-crystal samples. A further complication relates to the typically low transition temperature (*T*_c_) of these superconductors (SRO has a *T*_c_ below 1.5 K)^5^, which, in conjunction with the requirement of extreme purity, makes the fabrication of *p*-wave devices challenging. More recently, tunnelling studies^8^,^9^ have suggested that *p*-wave superconductivity can emerge on the surface of topological insulators coupled to *s*-wave superconductors, but the manipulation of these states for device applications is still an open issue. Triplet (*s*-wave) states have also been reported in spectroscopic experiments involving magnetically inhomogeneous *s*-wave superconductor/ferromagnet hybrids^10^,^11^,^12^.

Electrons in single-layer graphene (SLG) are predicted to condense to a superconducting state, either intrinsically by doping^13^,^14^,^15^,^16^,^17^,^18^,^19^,^20^ or by placing SLG on a superconductor with a BCS or a non-BCS pairing symmetry^21^,^22^. The resulting symmetry depends on the position of the Fermi energy (*E*_F_) with respect to the Dirac point. In particular, for *E*_F_ shifts up to 1 eV, a *p*-wave^15^,^16^ state is predicted. As the doping approaches the van Hove singularity (*E*_F_∼3 eV; ref. 19), a singlet chiral *d*-wave and triplet *f*-wave symmetry are also predicted^17^,^20^, whereas ref. 18 found dominant chiral *d*-wave superconductivity near van Hove doping and argued that weak coupling superconductivity for doping levels between half-filling and the van Hove density is of Kohn–Luttinger type and likely to be *f*-wave pairing for disconnected Fermi pockets. Reference 16^16^ predicted that a non-chiral *p*-wave symmetry is favoured for small nearest-neighbour repulsion (<1.1 eV), small onsite interaction *U* (∼8.4 eV) or large doping (above 10%), whereas the chiral *p*-wave state occurs as *U* or are increased or the doping level diminishes with respect to the aforementioned values (in pure SLG at half-filling *U* is ∼9.3 eV and is ∼5.5 eV; ref. 16). At low density (∼20%) and including next-nearest neighbour hopping, a chiral *p*-wave state can emerge^15^. Moreover, the possibility of spin-triplet *s*-wave pairing has been considered in bilayer graphene^23^.

Although intrinsic superconductivity in SLG has not been observed^24^, superconductivity has been induced by doping SLG with Li adatoms^25^, intercalating SLG sheets with Ca (ref. 26) or by placing SLG on a superconductor^27^. In the latter case, the intrinsic pairing potential for *p*- or chiral *d*-wave superconductivity can be enhanced theoretically^21^,^22^ to the point that a full transition to a superconducting state is triggered and manifested in the SLG superconducting density of states (DoS). Achieving *p*-wave or chiral *d*-wave superconductivity in SLG via a proximity effect would enable the fabrication of devices and allow the full investigation of how these symmetry states can be utilized in cryogenic technology related to spin transport with low dissipation. In addition, it would be attractive for applications to achieve *p*-wave superconductivity in SLG above 4.2 K.

The standard technique to probe electron pair symmetry involves measuring the voltage (*V*) bias dependence of the non-linear differential conductance (d*I*/d*V*−*V*) near the superconducting gap edge, which is proportional to the quasiparticle DoS in the tunnelling limit^28^. For a conventional superconductor with *s*-wave (zero spin) pairing, d*I*/d*V* diminishes below the gap edge in the tunnelling limit. In contrast, unconventional *p*- and chiral *d*-wave symmetries result in zero-energy quasiparticle excitations and surface bound states^29^, which manifest themselves in the tunnelling spectra as subgap structures in the DoS, including V-shaped gaps or strong zero-bias conductance peaks (ZBCPs)^30^,^31^,^32^,^33^.

Tonnoir *et al*.^27^ locally probed the superconducting DoS in SLG on the *s*-wave superconductor Re by scanning tunnelling microscopy (STM). They inferred induced superconductivity in SLG from the observation of a gapped DoS that matched the underlying layer of Re (*s*-wave). The absence of a subgap structure and, therefore, unconventional superconductivity, may indicate a modification of the SLG band structure^34^,^35^ due to the high carrier density of Re (*n*_e_∼4.5 × 10^23^ cm^−3^) resulting in significant charge transfer.

An alternative approach to ref. 27 is to place SLG on an oxide cuprate superconductor as the lower carrier concentrations of these materials (∼10^20^ cm^−3^; ref. 36) will reduce unwanted effects due to doping, such as band structure modifications. However, most oxide-based superconductors have a complex DoS due to their anisotropic superconducting order parameters^30^,^31^,^32^, which will necessarily complicate the analysis of the SLG DoS spectra, unless care is taken.

Pr_2−*x*_Ce_*x*_CuO_4_ (PCCO) shares similarities with other high-temperature oxide superconducting materials, such as YBa_2_Cu_3_O_7_ (YBCO). In particular, it has a *d*-wave symmetry, albeit with a much longer coherence length (ξs) of ∼30 nm (ref. 37) compared with that of YBCO of ∼2 nm (ref. 37). Tunnelling studies on hole-doped (*x*<0.13) PCCO (ref. 38) reveal anisotropic superconducting DoS consistent with *d*-wave symmetry with enhancements of the conductance at zero energy as observed on hole-doped YBCO (ref. 39). In contrast, for electron-doped PCCO (*x*>0.13), which is the case considered in our experiment where *x*=0.15 in PCCO, the superconducting DoS is found to be isotropic^37^,^40^ with a gapped DoS for all tunnelling directions. This is due to the electron-doped PCCO being in the dirty limit^40^ meaning *ξ*_s_>*l*_e_, with *l*_*e*_ being the electron mean free path.

Here we report a scanning tunnelling spectroscopy (STS) investigation of SLG placed on the electron-doped cuprate superconductor PCCO to probe the possibility of inducing unconventional superconductivity in SLG such as *p*-wave^15^,^16^ via a superconductor proximity effect. Our results reveal modifications of the superconducting DoS in SLG compared with the underlying PCCO at 4 K: a mixture of V-shaped gaps, strong ZBCPs and split ZBCPs are observed, depending on the position of the STM tip. A theoretical analysis indicates that the subgap structures match *p*-wave superconductivity with the different symmetry components of the *p*-wave state depending on the surface orientation of the underlying PCCO.

## Results

### PCCO characterization and Raman spectroscopy of SLG/PCCO

**Figure 1:**
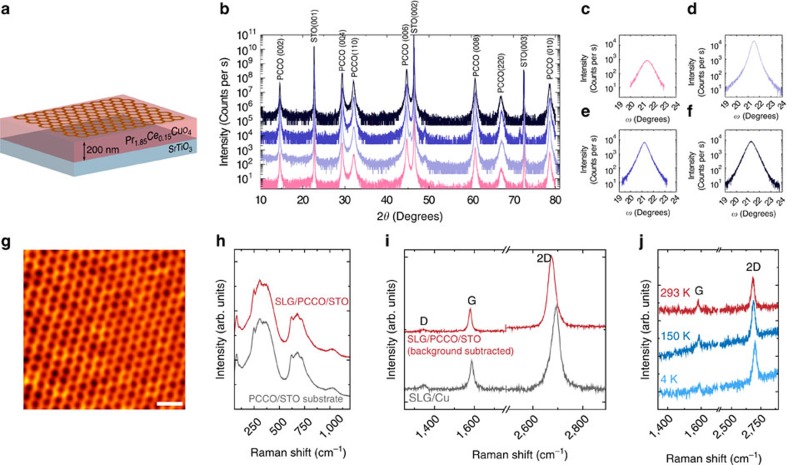
Characterization of SLG on electron-doped PCCO. (**a**) Schematic of SLG (hexagonal lattice) on PCCO/STO. (**b**) High-angle X-ray diffraction data on four control films of 5 mm × 5 mm PCCO/STO deposited using identical growth conditions (curves are vertically offset for clarity). (**c**–**f**) Rocking curves (omega scans) of the (006) PCCO diffraction peaks from the same samples investigated in (**b**) with matching colours showing a FWHM of 0.85° (**c**), 0.42° (**d**), 0.51° (**e**) and 0.61° (**f**). (**g**) STM topography map showing SLG on PCCO/STO measured at 4.2 K (scale bar in **g** has a length of 0.5 nm). (**h**) Raman spectra at 514.5 nm of PCCO as-grown on STO (grey) and following transfer of SLG (red) at 293 K. (**i**) Raman spectra at 514.5 nm of SLG as-grown on Cu (grey) and following transfer onto PCCO/STO (red) measured at 293 K. The PCCO/STO background is subtracted to allow identification of D, G and 2D peaks. (**j**) Raman spectra at 514.5 nm of SLG on PCCO/STO, after background subtraction, at 293 K (red), 150 K (blue) and 4.2 K (light blue).

The PCCO (200-nm-thick) is grown by pulsed laser deposition on (001) oriented SrTiO_3_ (STO) (see ‘Methods') and has a near-bulk superconducting transition of 20.5 K (see Fig. 1a and Supplementary Fig. 1a). High-angle X-ray diffraction performed on control PCCO films grown in the same conditions as those measured in this study by STM (Fig. 1b and Supplementary Fig. 1b) reveals poor *c*-axis texturing with strong peaks from the (110) family of planes. In particular, the rocking curves of the (006) diffraction peak of the PCCO films show a spread in the full width at half maximum (FWHM) in the range 0.42°–0.85° (Fig. 1c–f), which are large values for perfect (001) texturing. These values are comparable to the FWHMs of the diffraction peaks of the (110) family of planes (with differences typically of ∼0.3° for neighbouring peaks belonging to the two families of planes) as shown in Supplementary Fig. 1c–f, which confirms that the PCCO surface is a mixture of (100) and (110) planes. This is in agreement with previous studies^38^,^40^ which have shown that secondary (110) phases would always form during the PCCO growth.

SLG is grown on Cu by chemical vapour deposition (CVD), as described in ref. 41, and transferred onto PCCO/STO, following the procedure in ref. 42. The analysis of the Raman spectrum acquired on as-grown graphene on Cu (Fig. 1i, grey curve) shows that the 2D peak can be fitted with a single Lorentzian with a position, Pos (2D), ∼2681, cm^−1^ and FWHM(2D) ∼37 cm^−1^, indicating SLG (ref. 43). The G peak position, Pos(G), and FWHM(G) are 1,587 cm^−1^ and 20 cm^−1^, respectively; and the intensity ratio, *I*(2D)/*I*(G), and area ratio, *A*(2D)/*A*(G), are 2.9 and 5.6, respectively. These indicate a doping of ∼200 meV for SLG on Cu at room temperature^44^,^45^,^46^. The spectrum shows a small D peak with *I*(D)/*I*(G)=0.15, corresponding to a defect concentration of *n*_D_∼3.8 × 10^10^ cm^−2^ (refs 45, 47). A compressive biaxial strain of 0.07% is also estimated from the Raman analysis. To compare the SLG quality before and after transfer, the background signal of PCCO on STO is measured under identical conditions (see Fig. 1h and ‘Methods' section) and subtracted after normalization to the intensity of the Raman peak of STO at ∼300 cm^−1^. After transfer, Pos(2D) and FWHM(2D) are ∼2681, cm^−1^ and 37 cm^−1^, Pos(G) and FWHM(G) are 1,581 cm^−1^ and 17 cm^−1^, while *I*(2D)/*I*(G) and *A*(2D)/*A*(G) are 3.4 and 7.1, respectively (Fig. 1i, red curve). This implies a reduction in doping to <100 meV (refs 44, 45, 46), with *I*(D)/*I*(G) ∼0.09, corresponding to *n*_D_∼2.2 × 10^10^ cm^−2^. Similar values of defect concentration and strain for as-grown SLG on Cu and after its transfer onto PCCO imply homogeneous SLG on PCCO.

To assess doping and sample quality in the same temperature range used for the superconductivity studies, Raman measurements are also performed between 4.2 K and room temperature (Fig. 1j and Supplementary Fig. 2a). The low-temperature Raman data indicate that doping remains below 100 meV and that there are no structural changes compared with room temperature.

### Local density of states measurements on SLG/PCCO

**Figure 2:**
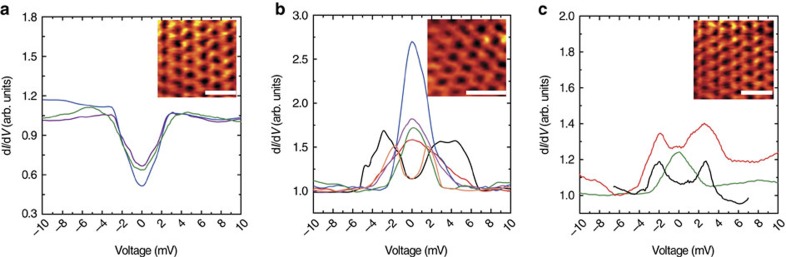
STM differential conductance versus bias-voltage spectra on SLG/PCCO/STO at 4.2 K. (**a**–**c**) Typical proximity-induced V-shaped gaps (**a**), ZBCPs (**b**) and split ZBCPs (**c**). Different colours in **a**–**c** are used to distinguish between spectra recorded in different sample areas. Insets in **a**–**c** show the typical topography of a sample area where the corresponding spectra in **a**–**c** are obtained (the scale bars in the insets have a length of 0.5 nm).

Using STM we locally measure d*I***/**d*V*−*V* spectra of SLG on PCCO at 4.2 K and correlate these to surface topography (Fig. 1g). The most predominant superconducting-related spectra show either V-shaped gaps (Fig. 2a) or a subgap structure, including ZBPCs and split ZBCPs (Fig. 2b,c). Additional supporting data are shown in Supplementary Fig. 3. V-shaped gaps are observed in about 45% of the scans, while in all the other areas we observe either ZBCPs (∼30%) or split peaks (∼25%). In the normal state none of these spectral features is observed, which rules out the possibility that these are due to electronic inhomogeneity in the sample, since this should give rise to the same features independently of temperature. In some areas, in all of which the SLG local topography cannot be properly resolved, single-electron tunnelling spectra are measured in the superconducting state (Supplementary Fig. 4); such features persist above *T*_c_ (at least up to 50 K), as also shown in Supplementary Fig. 4.

The evolution of superconducting-related spectral features is also studied as the sample is warmed up above its superconducting transition. All subgap features including ZBCPs and split ZBCPs are suppressed as the sample is warmed up (Supplementary Fig. 5) giving spectra that are either consistent with lightly doped SLG with *E*_F_ within 100 meV from the Dirac point^48^, or to structureless (flat) spectra, or single-electron tunnelling effects. The latter two appear in <10% of the total scanned area, where no superconducting-related features are observed below *T*_c_ and the STM topographic images do not exhibit a clear SLG structure. This shows that the proximity takes place only in regions that structurally and electronically conform to the SLG behaviour. Whilst proving that the V-shaped gaps, ZBCPs or split peaks are related to superconductivity, the absence of such structures (particularly conductance peaks) above the superconducting transition temperature, rules out spurious effects from defects (for example magnetic impurities) or structural inhomogeneity that may cause Kondo scattering and thus enhancements of the DoS, as such features would also be present in the normal state. Further support for this conclusion comes from the fact that ZBCPs are found (below *T*_c_) only in regions where the STM images show clear SLG topography and not in defected regions. We also measure the evolution of ZBCPs in an applied out-of-plane magnetic field (Supplementary Fig. 6), and their magnitude is always found to decrease, with no splitting ever observed, which is also inconsistent with a Kondo effect. This effect of magnetic field is similar to those observed in tunnelling spectroscopy studies of proximity-induced *p*-wave superconductivity in Bi_2_Se_3_ (ref. 9) and odd-parity topological superconductivity in Cu_x_Bi_2_Se_3_ (ref. 49).

### Control experiments on bare PCCO and Au/PCCO

**Figure 3:**
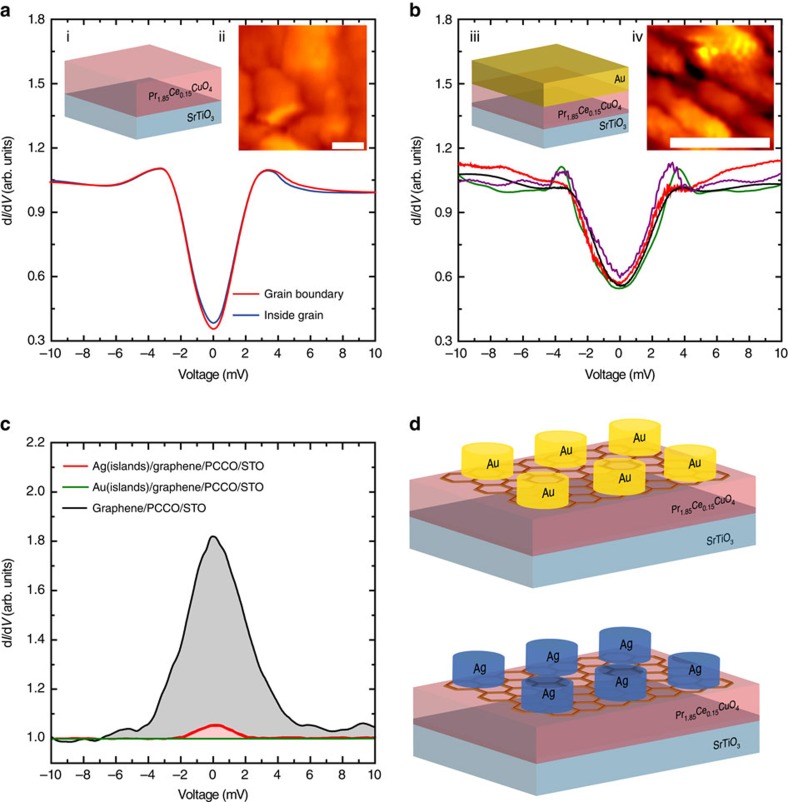
STM differential conductance versus bias-voltage spectra on control samples of PCCO/STO and Au/PCCO/STO at 4.2 K. (**a**) Typical STM spectra at 4.2 K for PCCO/STO with schematic of the sample structure (inset i) and STM topography (inset ii; the scale bar has a length of 0.5 nm). (**b**) Typical STM spectra at 4.2 K for Au/PCCO/STO (Au is 10-nm-thick) with schematic of the sample structure (inset iii) and STM topography (inset iv; the scale bar has a length of 0.5 nm). (**c**,**d**) Average ZBCP amplitudes for SLG/PCCO/STO (black shaded curve) and Au (green shaded curve) and Ag (red shaded curve) microislands on SLG/PCCO/STO (**c**) with corresponding schematics shown in (**d**) where the hexagonal lattice represents SLG.

Control samples of bare PCCO are also investigated, but no subgap structure or V-shaped gaps are observed (Fig. 3a), even at facets that expose the nodal *ab*-plane. The only DoS features we can observe are smeared BCS-like gaps, consistent with previous STS experiments on electron-doped PCCO (refs 37, 40). We investigate the effect of substrate choice on the superconductor proximity effect by fabricating SLG/PCCO on (001) LaAlO_3_ (LAO) and observe similar results to STO (Supplementary Fig. 7).

The ZBCPs on SLG/PCCO might be related to the penetration of the anisotropic components of PCCO that are masked in bare PCCO, but which may appear in SLG due to its long electron mean free path (∼100 nm near the Dirac point)^50^ and spin diffusion length (>1 μm) (ref. 51) being much longer than the coherence length in PCCO of ∼30 nm (ref. 37). These are important parameters to consider, since the superconducting condensate is induced in the entire graphene plane. To check this, we replace SLG with Au and fabricate Au/PCCO/STO films (Fig. 3b), where a 10-nm-thick polycrystalline Au layer is deposited *in-situ* without breaking vacuum by pulsed laser deposition, and perform STS. Au is chosen since it has an electron mean free path ∼30 nm at room temperature^52^. The topography maps of Au/PCCO/STO (Fig. 3b) reveal a low surface roughness (∼1 nm over a 1 μm^2^ area; Supplementary Fig. 8), and the corresponding tunnelling spectra on Au show superconducting gaps with no subgap structure, thus supporting our claim that the subgap structures in SLG/PCCO are related to SLG and not the underlying PCCO. We note that the gapped spectra on Au/PCCO are shallower than those of bare PCCO (Fig. 3a,b), but qualitatively similar, suggesting that Au is fully superconducting.

### Tuning the superconducting proximity effect in SLG/PCCO

To further confirm our claim that the spectral features on SLG/PCCO are related to a superconductor proximity effect in SLG and not the underlying PCCO, we also measure spectra on SLG covered with evaporated microislands (10 μm in diameter and 30 nm in height) of either Ag or Au (Fig. 3c,d). Unlike metals, such as Pd, which have a stronger binding interaction with SLG (ref. 34), the low binding energy of Au and Ag (0.04 eV per carbon atom^34^) results in a reduced modification of the SLG band structure^34^. Ag (Au) has a work function of 4.4 eV (5.2 eV) which is lower (higher) than the work function of SLG (∼4.6 eV; ref. 34). Therefore, if a superconducting proximity effect occurs on SLG/PCCO, this should be stronger near a Ag microisland than a Au one, since Ag (Au) acts as a donor (acceptor) of electrons in SLG, with a doping profile that can extend up to hundreds nanometre from the microisland step edge^53^. The tens of d*I***/**d*V*−*V* spectra acquired on these samples show that pronounced ZBCPs are observed near and even on the Ag microislands, but no ZBCPs are ever observed near or on Au microislands, despite the well-defined SLG topography (some representative spectra are shown in Fig. 3c and in Supplementary Figs 9 and 10). This implies that the Au islands reduce the SLG electron density to the extent that the superconducting proximity effect for SLG on PCCO is suppressed. We also note that these data indicate that possible doping inhomogeneities can only affect the size of the unconventional superconducting features (even to their suppression, along with the proximity effect altogether), but do not affect the unconventional nature of the proximity-induced superconductivity in SLG.

### Calculation of the superconducting DoS in SLG/PCCO

**Figure 4:**
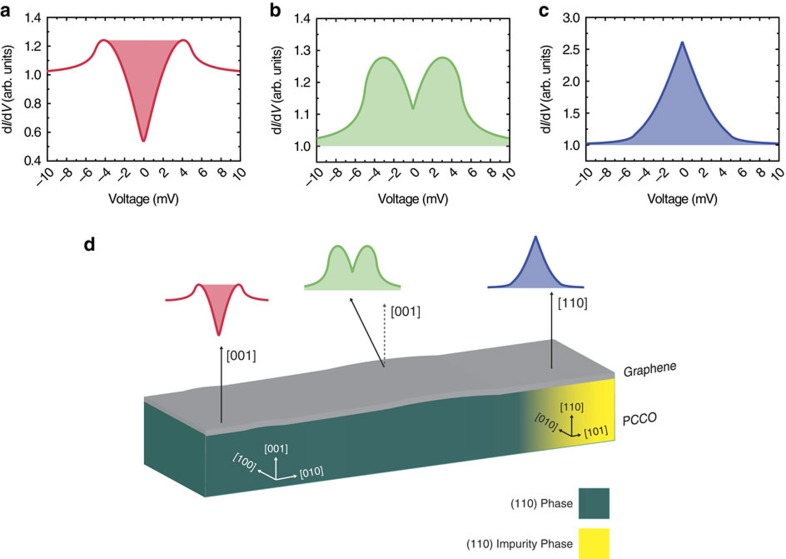
Theoretical proximity-induced superconducting DoS in SLG on PCCO. (**a**–**d**) The plots show that the projections of the *p*-wave induced symmetries in SLG are dependent on the crystal orientation of the underlying PCCO. This is clearest in (**d**) which illustrates the relationship between the observed DoS on SLG and the plane normal to the underlying PCCO: on flat regions oriented along the [001] crystallographic direction, the DoS corresponds to *p*_*y*_-wave or antinodal 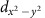-wave with a strong tunnelling barrier (red curve in **a**); rough areas expose a mixture of normal planes oriented between the [001] and [110] crystallographic directions, where the DoS corresponds to *p*_*y*_-wave with a moderate tunnelling barrier (green curve in **b**); the DoS corresponding to the *p*_*x*_-wave is projected on the phase oriented along the [110] crystallographic direction (blue curve in **c**).

We now consider the spatial variations in the superconducting DoS that we observe on SLG/PCCO (Fig. 2 and Supplementary Fig. 3). These indicate local variations in the proximity effect due to a combination of PCCO faceting and changes in the PCCO surface that is a mixture of different PCCO planes. Fig. 4 plots the theoretical DoS on SLG as a function of the STM orientation relative the graphene surface, which varies due to the PCCO faceting.

These are calculated by applying the model in refs 22, 54, which predicts an effective *p*-wave pairing at the Dirac points in SLG on a *d*-wave superconductor and involves solving a tight-binding Hamiltonian for SLG assuming that the tunnelling between SLG and the STM tip obeys an extended Blonder–Tinkham–Klapwijk (BTK) theory^30^,^55^. Following refs 22, 54, we write the Hamiltonian in a band-basis where *c*_q_ is a Fermion-operator in the conduction band and *d*_q_ is a Fermion-operator in the valence band:


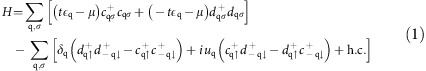


In equation (1) we define the normal-state band dispersion as *ɛ*_q_=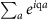 where the sum is over nearest-neighbour vectors *a, t* is the hopping parameter, *μ* is the chemical potential, while *δ*_q_ and *u*_q_ are associated to the superconducting order parameter. The above Hamiltonian can be diagonalized and yields eigen values:


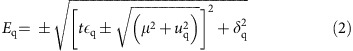


Since the shift in Fermi level (*E*_F_) in SLG (below 100 meV in SLG on PCCO at 4.2 K) is much larger than the superconducting order parameter (*Δ*∼5 meV for PCCO), *u*_q_ in equation (2) can be neglected and we are left with an effective superconductor with gap *δ*_q_. It is this gap that acquires a *p*-wave symmetry near the Dirac points when *d*-wave superconducting correlations are induced in SLG^22^,^54^. This is due to the fact that the transport properties of SLG are determined by its behaviour near the Dirac points for doping levels comparable to that in our study (<100 meV) and, as explained in refs 22, 54, a *d*-wave symmetry in the full Brillouin zone corresponds to a *p*-wave symmetry in the vicinity of the Dirac points at K and K'.

In this regime, where the shift in *E*_F_ in SLG is much larger than *Δ*, a simplified model for tunnelling between SLG and the STM-tip based on BTK theory can be adopted to account for *p*-wave pairing. This is a commonly used modelling tool for tunnelling measurements with unconventional superconductors^56^,^57^,^58^. The procedure consists in setting up the wavefunctions on SLG and on the normal side of the tunnelling barrier, the strength of the latter being characterized by a dimensionless number *Z*. The *Z* parameter describes the strength of the tunnel barrier between SLG and the STM tip, that is, the barrier is effectively the vacuum in between. The *Z* value affects the DoS spectra as it is related to the ratio between Andreev reflections and quasiparticle tunnelling (*Z*=0 corresponds to a perfect interface with zero resistance, whereas *Z*>>1 corresponds to a barrier with high tunnel resistance). The *Z* value can be controlled in STS via the current and bias-voltage settings (see below), which governs the tip-sample distance in the case of an ideally homogeneous sample. However, in our experiment, it is realistic to expect spatial variations in *Z* (for a given current-bias setting) since the STM tip approaches the graphene surface at slightly different distances at different locations, due to variations in the surface plane of the underlying PCCO and local variations in graphene-PCCO connectivity, which is hence accounted in our theoretical model.

The theoretical DoS in Fig. 4 are obtained with *Δ*=5 meV, *δ*_q_=0.1 meV and *Z* varying between 0.8 and 1.7, and they show that the surface orientation of PCCO sensitively controls the DoS, due to the projection of the *p*_*x*_ and *p*_*y*_ symmetry components of the *p*-wave state. Importantly, in this *Z* regime, enhanced conductance at zero bias, and therefore the ZBCPs, cannot be related to Andreev reflections unless the order parameter is unconventional, meaning it does not have an *s*-wave symmetry. In addition, the sharp structure of the peaks that we observe experimentally (Fig. 2 and Supplementary Fig. 3) can be accounted for only by sign-changing order-parameter symmetry, such as *p*-wave.

## Discussion

The modifications of the superconducting DoS on SLG/PCCO/STO (Fig. 2 and Supplementary Fig. 3) compared with PCCO/STO (Fig. 3a) or Au/PCCO (Fig. 3b), in particular the ZBCPs and split peaks, indicate the induction of *p*-wave^15^,^16^ or chiral *d*-wave pairing in SLG^20^. However, chiral *d-*wave requires dominant electron-electron repulsion in SLG at much higher doping levels (up to 3 eV; ref. 20) than here (∼100 meV). In our low doping regime, *p*-wave pairing should theoretically dominate^15^,^16^,^22^ with spectral features in the DoS that match our findings in Fig. 2.

We also exclude other unconventional pairing symmetries that may be compatible with the spectral features we observe, such as *f*-wave pairing as suggested in ref. 59, since this would require a buckling in the honeycomb lattice, which we do not expect for SLG. Another possibility, however, would be to consider the emergent order parameter symmetry near the Dirac points in the presence of f-wave pairing^17^,^18^ in the Brillouin zone to determine if this could also provide zero-energy states.

Our theoretical model shows that an induced *p*_*x*_-wave state is manifested in the SLG DoS as a ZBCP (Fig. 4c), while an induced *p*_*y*_-wave state manifests as a split-ZBCP (Fig. 4b). If tunnelling occurs along the *c*-axis of the PCCO (along [001]), a V-shaped gapped DoS consistent with *p*_*y*_ or antinodal 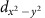 symmetry is observed (Fig. 4a).

The theoretical analysis is consistent with the distribution in the DoS spectral features that we measure on the SLG/PCCO (∼45% V-shaped gaps, ∼30% ZBCPs and ∼25% split peaks). Although it is not possible to determine the orientation of the underlying PCCO below the STM tip during measurements, the X-ray data in Fig. 1b indicate that the surface of PCCO is a mixture of the (001) and (110) family of planes, and therefore the DoS according to our theory should be a mixture of V-shaped gaps (occurring for *c*-axis tunnelling), ZBCPs or split peaks (occurring for tunnelling from the *ab*-plane). The surface roughness of the PCCO will also expose different crystal orientations. Therefore, the spectroscopic features on SLG/PPCO will be related to a combination of PCCO crystal structure and surface roughness. Figure 4 also shows that the V-shaped DoS calculated for SLG/PCCO does not reach zero, which is consistent with our experimental observations (Fig. 2a and Supplementary Fig. 3), but also with previous tunnelling experiments on PCCO even at temperatures much lower (1.8 K) than our measurement set-up of 4.2 K (ref. 40).

Local variations in the Z parameter can determine the amplitude of subgap features (ZBCPs or split peaks) and the depth of V-shaped gaps, as shown in Supplementary Fig. 11. Although Z values can be changed intentionally by varying the set current and set bias-voltage values (before disabling the feedback loop and acquiring a d*I*/d*V* spectrum), care was taken to work with low set currents (∼0.1–0.3 nA) and bias voltages just above the gap region (8–10 mV), yielding junction resistances ∼5 × 10^7^ Ω, much larger than the quantum resistance h e^−2^ (∼25.8 kΩ) and thus well within the tunnelling regime. In this regime, the overall spectral features (ZBCPs, split ZBCPs or V-shaped gaps) measured at a specific location on SLG/PCCO do not change upon varying the current and voltage set values, ruling out the possibility that these are related to single-electron tunnelling effects^60^. It should be noted, however, that the underlying orientation of the PCCO film and the degree of local PCCO-SLG electrical connectivity can also influence the tip-SLG distance and hence the *Z* value (for a given current-bias setting). We exclude that variations in the spectral features are due to defects in SLG, since STM images do not reveal any structural inhomogeneity in SLG and no damage after transfer (consistent with the Raman analysis), and, in particular, no defects are observed in regions where the spectra are acquired. Further, spectral anomalies disappear in the normal state.

Finally, we point out that in ref. 61 subgap features in the DoS including ZBCPs and split zero-bias peaks could be obtained via Andreev bound states, formed under a small Pb slab coupled to SLG. Such a system effectively constituted a quantum dot coupled to superconducting leads. Here, the local and random doping inhomogeneities in SLG on PCCO are not likely to induce well-defined quantum dots, where confinement would be further suppressed by Klein tunnelling, and thus cannot account for the subgap features seen in our STM data.

Although our analysis is consistent with the triggering of an unconventional *p*-wave order parameter in SLG, we cannot determine the exact symmetry of the *p*-wave state. Since the DoS spectra look qualitatively different from those reported for a chiral spin-triplet *p*-wave state in a topological superconductor^8^,^9^, we believe our results to be consistent with an effective *p*-wave order parameter emerging at the Dirac points in SLG as a result of Andreev bound states induced by the proximity coupling with the *d*-wave pairing potential in PCCO (ref. 62). The results therefore suggest the exciting possibility of creating *p*-wave superconducting devices on SLG in which quantum coherent electron states, gate tunability and spin/charge could be addressed in the superconducting state.

Measurements of quasiparticle interference modulations^63^ could provide further insights into the pairing symmetry induced in SLG on PCCO. Our results suggest that the superconducting pairing of the *p*-wave state in our system is spin-singlet, which means that the Andreev bound states providing the zero-energy peak seen in the STM data can be interpreted as an odd-frequency spin-singlet odd-parity state^64^,^65^. Interfacial spin–orbit coupling due to broken inversion symmetry at the PCCO/SLG interface may also in principle induce a triplet component in the system^66^; however, the induction of a triplet component would necessary require significant spin–orbit coupling at the SLG/PCCO interface, unlikely here due to the low atomic numbers of the elements in PCCO and graphene.

## Methods

### PCCO film growth

A 200-nm-thick PCCO film is grown by pulsed laser deposition on (001) STO using a stoichiometric target fabricated by a solid-state reaction method from high purity Pr_6_O_11_ (99.9% purity), CeO_2_ (99.9% purity) and CuO (99.99% purity) powders. The growth is carried out at 820 °C by firing a Lambda Physik KrF excimer laser (*λ*=248 nm) on a rotating PCCO target with a pulse rate of 7 Hz and energy density of 1.5 J cm^−2^, after introducing 230 mTorr of N_2_O in a ultra-high vacuum chamber (base pressure of 10^−8^ mbar). To obtain an optimal superconducting transition (20.5 K; Supplementary Fig. 1a), the PCCO films are annealed *in situ*^67^ by evacuating the chamber immediately after growth and holding the substrate at 720 °C for 4 min. Films are then cooled to room temperature in a vacuum of 10^−5^ mbar within 2.5 h.

### Growth and transfer of SLG on PCCO

SLG is grown by CVD on a 35-μm-thick Cu foil loaded into a hot wall tube furnace, which is subsequently evacuated to a pressure ∼1 mTorr (ref. 41). The Cu foil is annealed in a hydrogen atmosphere (H_2_, 20 sccm) at 1,000 °C for 30 min to reduce the Cu oxide component and to increase the grain size^41^,^68^. SLG growth is then initiated by adding 5 sccm CH_4_ to the H_2_ flow. After 30 min, the substrate is cooled in vacuum (1 mTorr) to room temperature and then unloaded from the reactor.

CVD is chosen over micromechanical exfoliation^42^,^69^ so as to cover large areas (5 × 5 mm^2^) of PCCO with SLG. This is beneficial for a number of reasons: the localization and alignment of the sample is simplified (the scan size of our STM is limited to 1 × 1 μm^2^, and the optical contrast of SLG on PCCO does not allow for an easy identification of SLG); the large area allows us to fabricate large arrays of metallic islands (covering 2 × 2 mm^2^ of the sample surface); the measurements benefit from larger statistics and the presence of local defects can be avoided, thus not disturbing tunnelling-current measurements, which only require nm-size areas to be performed.

The CVD-grown SLG is then transferred onto 200-nm-thick epitaxial films of electron-doped PCCO on (001) STO. To do so, a 500-nm-thick layer of polymethyl methacrylate (PMMA) is spin coated onto the SLG/Cu sample. The PMMA/SLG/Cu stack is subsequently immersed in an aqueous solution of ammonium persulfate (APS) to etch the Cu foil^41^,^42^. The PMMA/SLG stack is then placed in de-ionized water to rinse off acid residuals and fished off the de-ionized bath using the PCCO substrate. Finally, the PMMA/SLG/PCCO sample is dried at room temperature and placed in acetone to remove the PMMA layer, leaving SLG on PCCO.

### Raman measurements

Raman spectroscopy is used to investigate the quality and uniformity of the as-grown SLG on Cu as well as to quantify the presence of defects and evaluate doping. Raman spectra are recorded at 514.5 nm using a Renishaw InVia spectrometer equipped with a Leica DM LM microscope and a × 100 objective (numerical aperture NA=0.85). Under these conditions the laser spot size is ∼1 μm. The laser power is kept at ∼500 μW to avoid any possible sample damage.

To assess the doping and quality of SLG as a function of temperature, in particular below the superconducting critical temperature of PCCO of 20.5 K, low temperature Raman measurements are performed between room temperature and 4.2 K (Supplementary Fig. 2a). These are carried out using an Oxford Instrument cryostat coupled with a Horiba LabRAM HR Evolution Raman spectrometer. An Olympus LUCPlanFL N × 40 objective with an aberration correction ring is used. This is adjusted to match the glass window thickness of the cryostat to enhance the signal-to-noise ratio.

Each sample is fixed to the holder in the cryostat with vacuum grease (Apiezon N Grease) and the cryostat is pumped to 10^−6^ Torr before measurements, to reproduce the same vacuum conditions used in the STM. The sample is cooled to 4.2 K using liquid helium and the temperature is raised stepwise with a programmable temperature controller. For each temperature, a Raman spectrum of SLG on PCCO is recorded as well as a reference spectrum on a bare PCCO film on (001) STO. To reveal the SLG contribution to the Raman signal, the spectrum measured on PCCO at a given temperature is subtracted from the corresponding one on SLG/PCCO.

### Data availability

The data set generated and analysed during this study are available for access at http://dx.doi.org/10.17863/CAM.6228.

## Additional information

**How to cite this article:** Di Bernardo, A. *et al*. *p*-wave triggered superconductivity in single-layer graphene on an electron-doped oxide superconductor. *Nat. Commun.* **8,** 14024 doi: 10.1038/ncomms14024 (2017).

**Publisher's note**: Springer Nature remains neutral with regard to jurisdictional claims in published maps and institutional affiliations.

## Supplementary Material

Supplementary Information Supplementary Figures 1-11 and Supplementary References
