# Corrigendum: *p*-wave triggered superconductivity in single-layer graphene on an electron-doped oxide superconductor

**DOI:** 10.1038/ncomms14817

**Published:** 2017-03-01

**Authors:** A. Di Bernardo, O. Millo, M. Barbone, H. Alpern, Y. Kalcheim, U. Sassi, A. K. Ott, D. De Fazio, D. Yoon, M. Amado, A. C. Ferrari, J. Linder, J. W. A. Robinson

Nature Communications 8: Article number: 14024; DOI: 10.1038/ncomms14024 (2017); Published: 01 19 2017; Updated: 03 01 2017

The present address for U. Sassi is incorrect in this Article. This author does not have a present address. The correct full affiliation details for this author are given below:

Cambridge Graphene Centre, University of Cambridge, Cambridge CB3 0FA, UK.

