# Supplementary material for: p-wave triggered superconductivity in single-layer graphene on an electron-doped oxide superconductor
Source: Nat Commun. 2017 Jan 19;8:14024. doi: 10.1038/ncomms14024 (PMC5253682; doi:10.1038/ncomms14024)
Supplement: Supplementary Information — Supplementary Figures 1-11 and Supplementary References [file ncomms14024-s1.pdf]

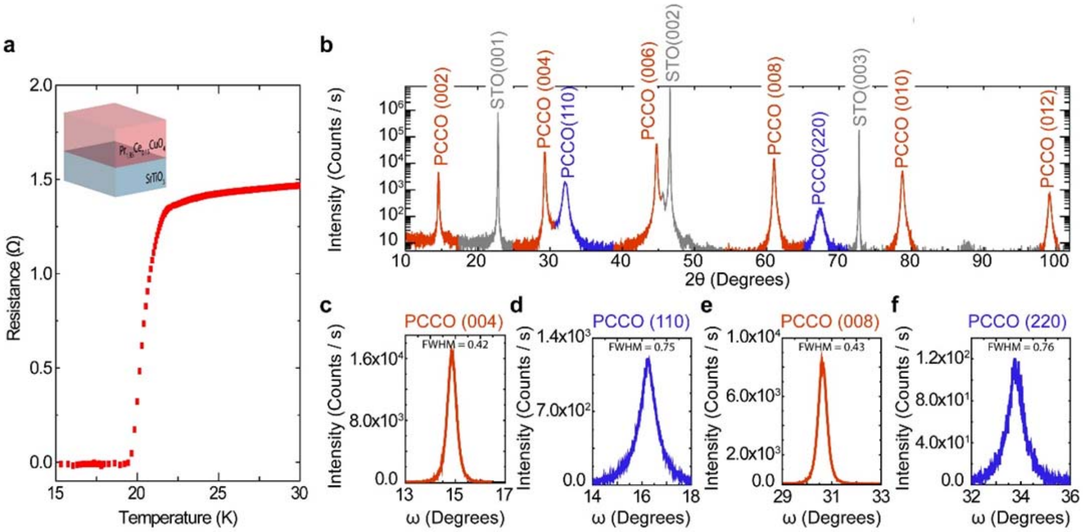

Supplementary Figure 1. Electronic transport and X-ray characterization of PCCO on STO. **a**, Resistance versus temperature (R-T) curve measured in a liquid helium Dewar using a four-point current-bias setup (10 μA) showing a superconducting transition of approximately 20.5 K. Inset: Schematic of sample structure. **b**, High angle X-ray diffraction data on a control film of 5 mm x 5 mm PCCO/STO deposited using identical growth conditions to the samples investigated by scanning tunneling microscopy. **c**, **d**, **e**, **f**, Rocking curves (omega scans) of the (004) (**c**), (006) (**d**), (008) (**e**), (220) (**f**) PCCO film diffraction peaks showing a full width at half maximum of 0.42° (**c**), 0.75° (**d**), 0.43° (**e**), 0.76° (**f**).

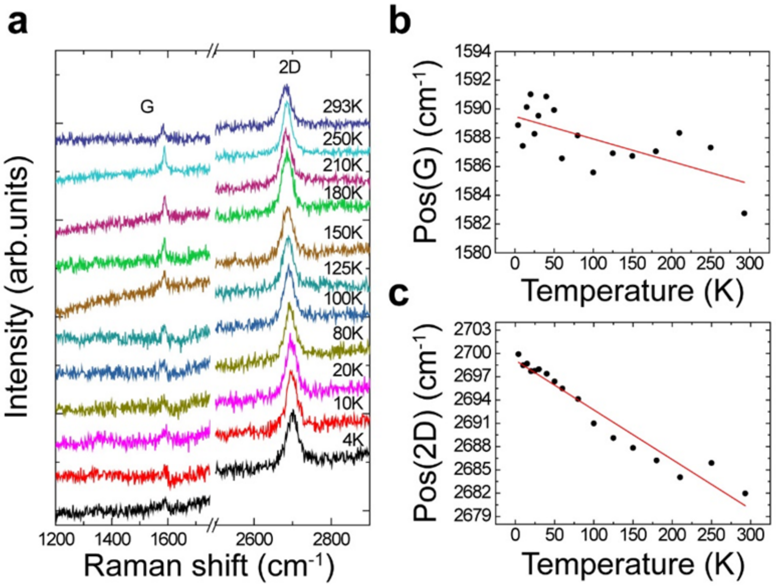

Supplementary Figure 2. Raman data on SLG/PCCO/STO as a function of temperature. **a**, **b**, **c**, Raman spectra at 514.5 nm as a function of temperature for SLG on PCCO, after subtraction of the PCCO on (001) STO background (**a**). Pos(G) (**b**) and Pos(2D) (**c**) as a function of temperature. The fitted Pos(G) and Pos(2D) show a linear dependence on temperature (**b** and **c**) with a slope of $\Delta Pos(G)/\Delta T = -0.016 \pm 0.003\ cm^{-1}/K$ and of $\Delta Pos(2D)/\Delta T = -0.064 \pm 0.004\ cm^{-1}/K$, respectively, consistent with temperature-induced anharmonic effects[1]. This as well as the other fitting parameters $I(2D)/I(G)_{4K} = 5.6$, $I(2D)/I(G)_{150K} = 3.4$, $I(2D)/I(G)_{293K} = 3.2$, $A(2D)/A(G)_{4K} = 13.7$, $A(2D)/A(G)_{150K} = 9.4$, $A(2D)/A(G)_{293K} = 7.1$, suggest that the doping level stays the same throughout the measurements, i.e. < 100 meV. In addition, no defects are introduced in SLG with temperature, as shown by the absence of a significant D peak[2].

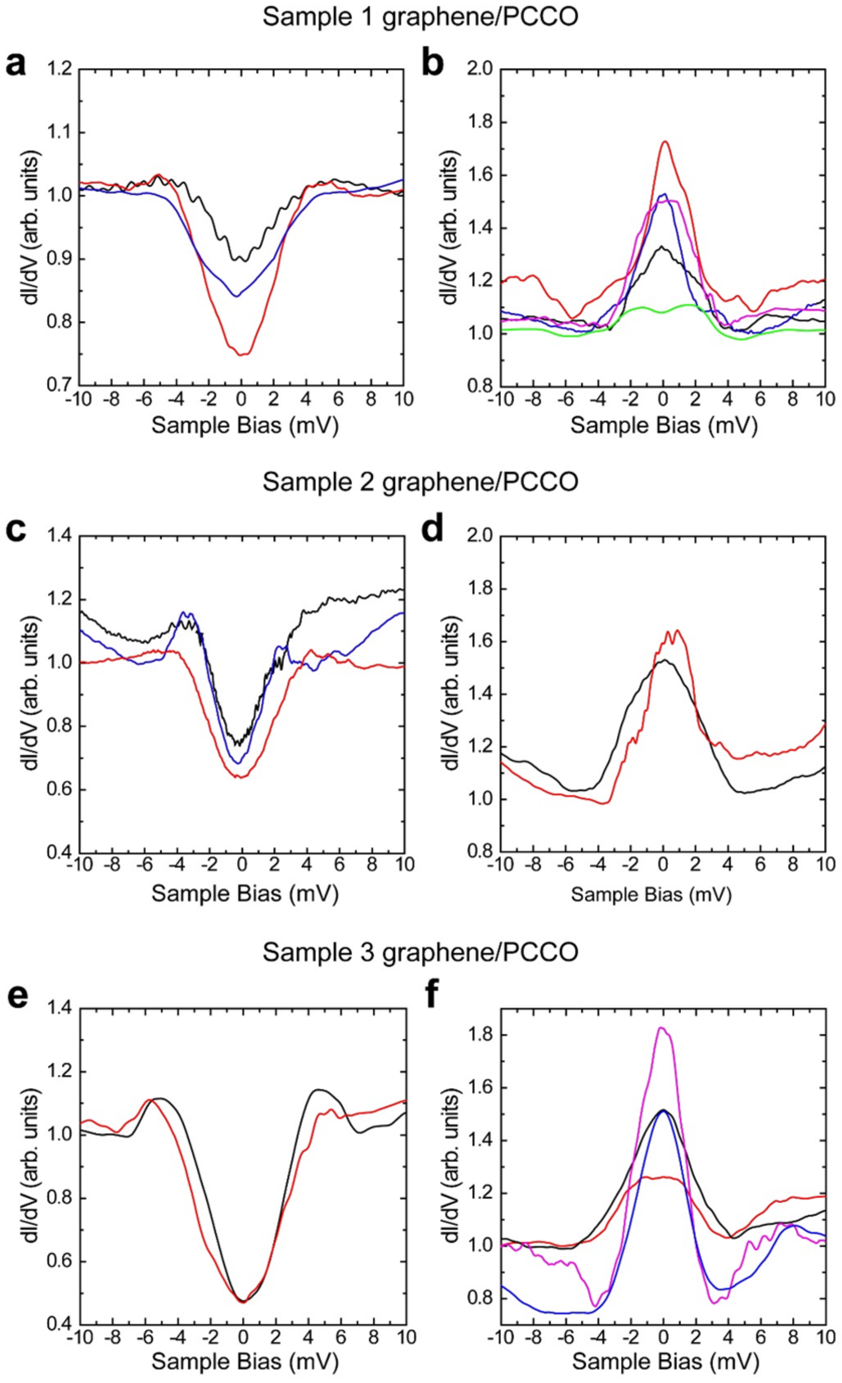

Supplementary Figure 3. Density of states measurements on SLG/PCCO/STO. a, **b**, **c**, **d**, **e**, **f,** Scanning tunneling differential conductance as a function of bias voltage at 4.2 K on different SLG/PCCO/STO samples, additional to those reported in Fig. 2 of the main text. For each sample, both V-shaped BCS gaps (**a**, **c** and **e**) and ZBCPs or split-ZBCPs (**b**, **d**, **f**) are observed, consistently with Fig. 2 and what discussed in the main text.

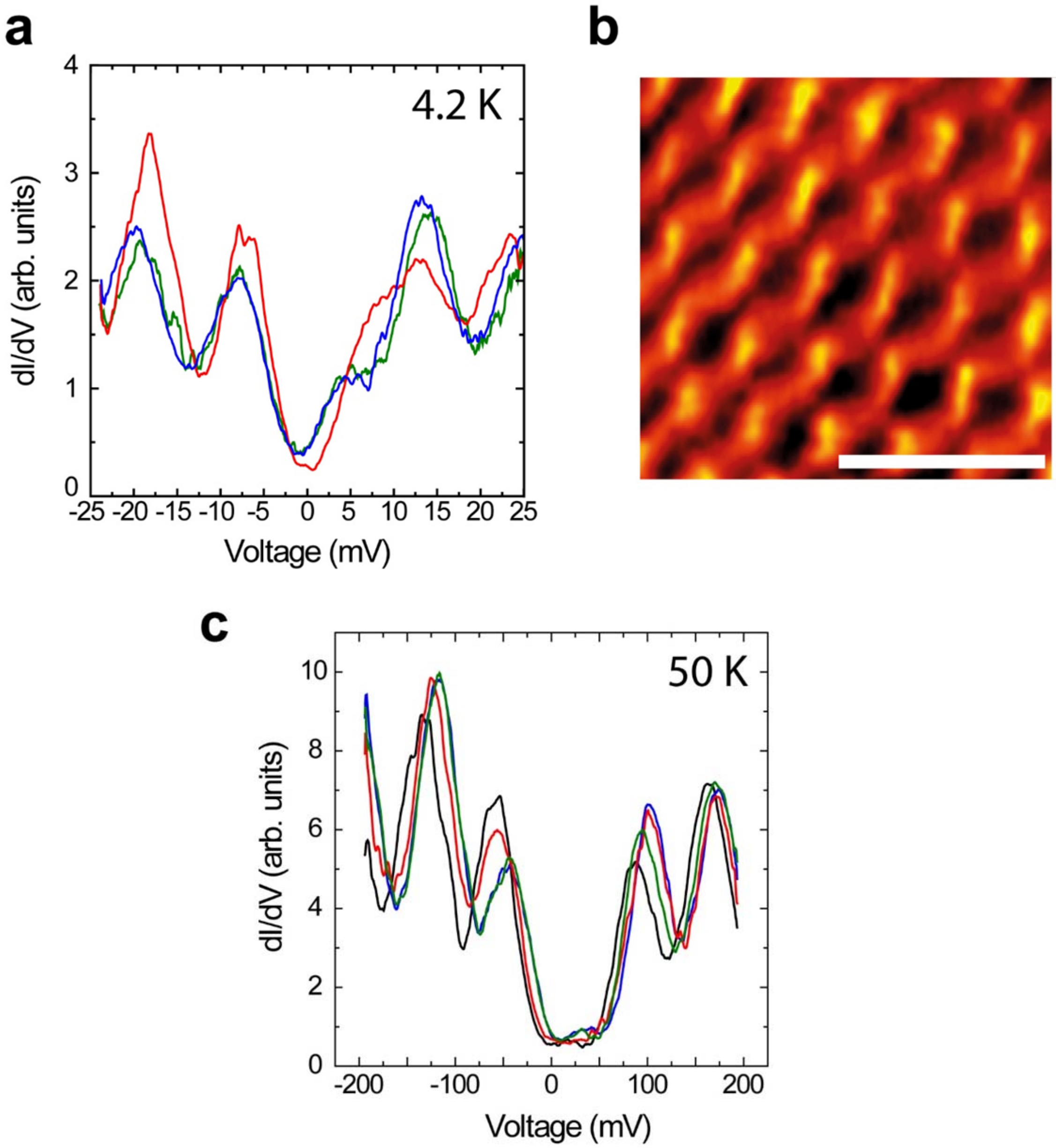

Supplementary Figure 4. Density of states measurements on SLG/PCCO/STO showing single-electron tunneling features. a, **b**, Scanning tunneling differential conductance spectra as a function of bias voltage measured at 4.2 K on SLG/PCCO/STO and showing single-electron tunnelling features (**a**). These are typically measured in areas where the SLG local topography cannot be resolved, as shown in (**b**) where the scale bar has a length of 0.5 nm. Such features were observed also above T_c. The spectra presented in (**c**) were acquired at 50K on another region, where the effective Coulomb island was smaller, giving rise to larger charging energy.

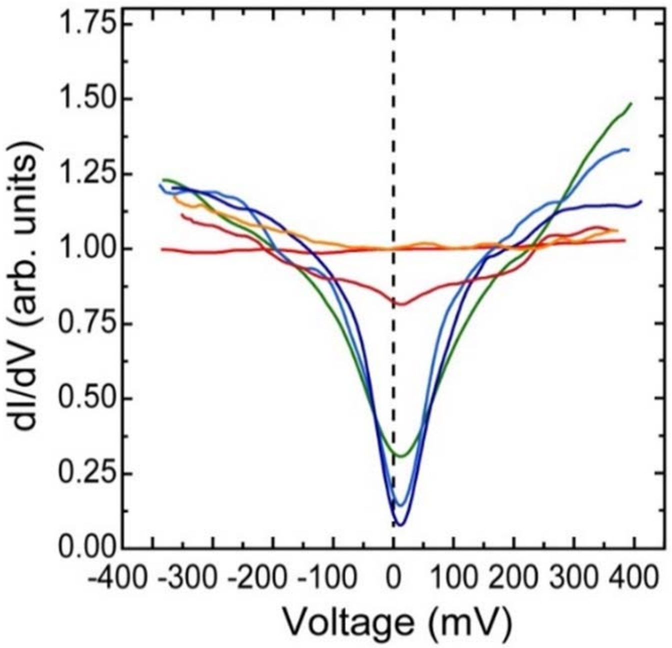

Supplementary Figure 5. Density of states measurements on SLG/PCCO on STO above the PCCO superconducting transition. Scanning tunneling differential conductance as a function of bias voltage at 50 (±5) K, i.e. above the PCCO superconducting critical temperature (T_c), on SLG/PCCO/STO areas where V-shaped gaps and ZBCPs are measured below T_c (blue and green curves) and in areas where no superconductivity-related spectra features are found below T_c (orange and red curves) and the topography did not show clear SLG structure.

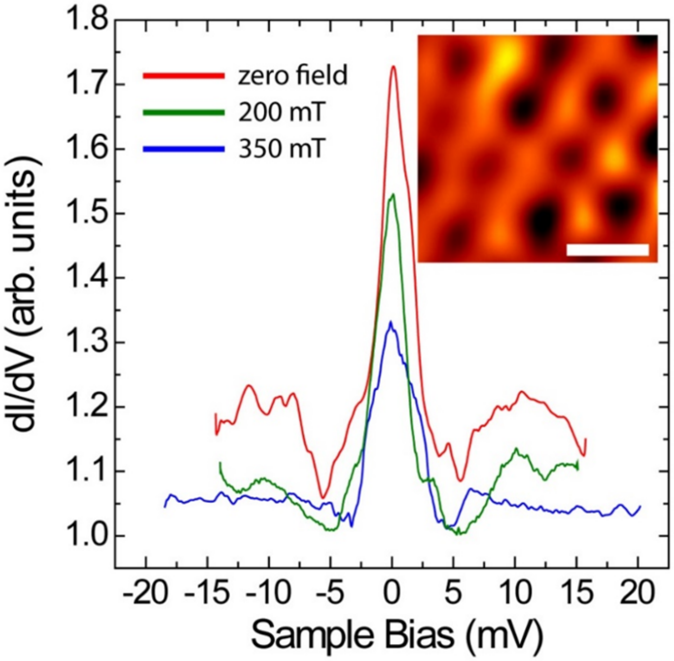

Supplementary Figure 6. Magnetic field dependence of a zero-bias conductance peak on SLG/PCCO/STO. Scanning tunneling differential conductance as a function of bias voltage measured at 4.2 K on SLG/PCCO/STO showing a zero-bias conductance peak and its evolution in an applied out-of-plane magnetic field. The tunneling spectra were all acquired on the same spot, which

belongs to the sample area with SLG local topography shown in the inset (the scale bar has a length of 0.3 nm).

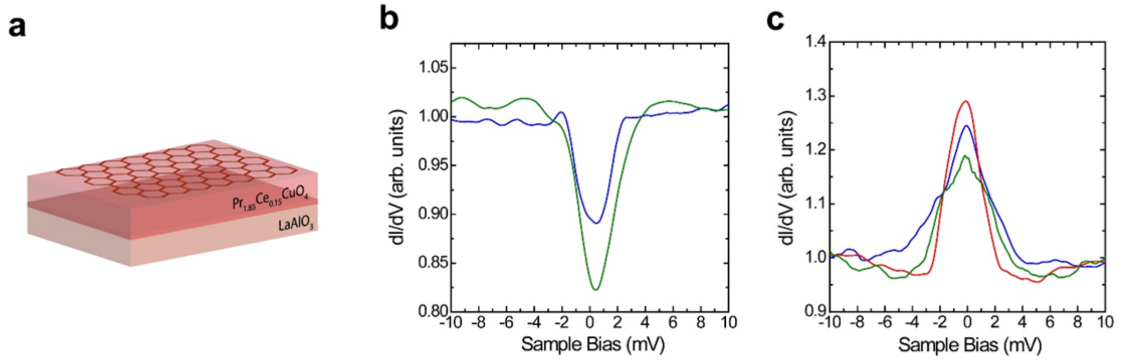

Supplementary Figure 7. Density of states measurements on SLG/PCCO on LAO substrate. a, **b**, **c**, Scanning tunneling differential conductance as a function of bias voltage measured at 4.2 K on SLG/PCCO on (001) oriented LAO substrate (the hexagonal lattice represents SLG). Schematic of sample structure (**a**), V-shaped gaps (**b**), and ZBCPs (**c**).

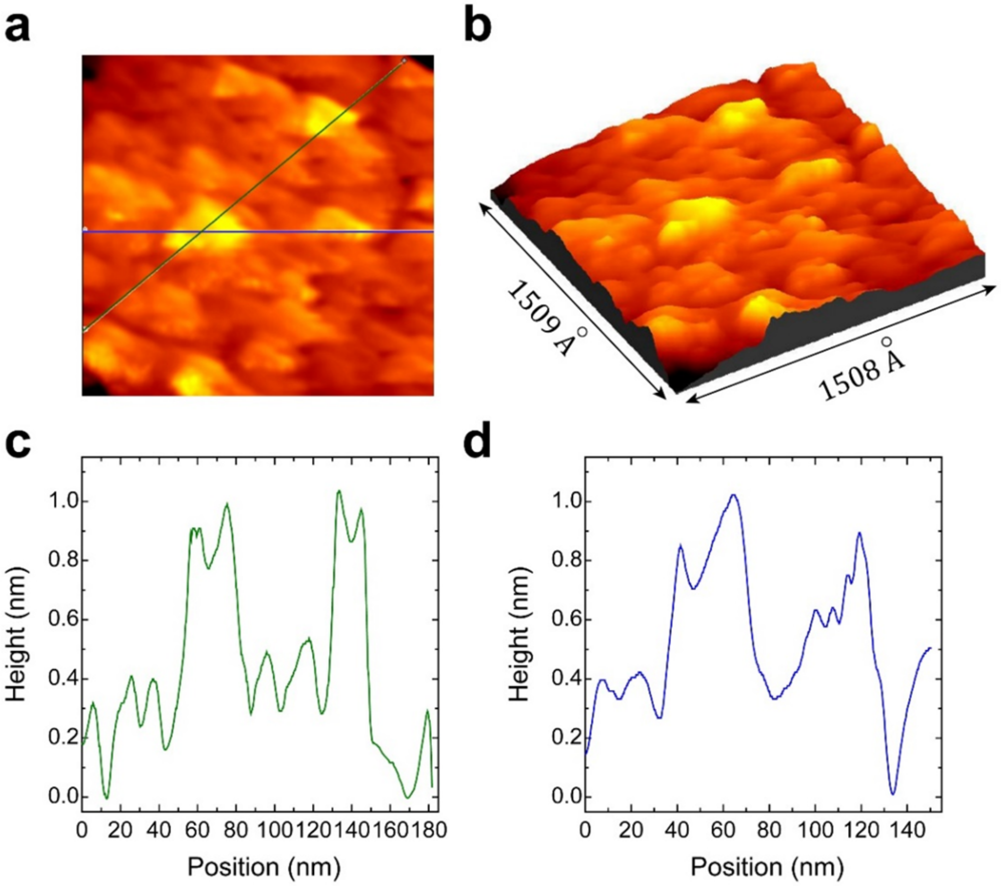

Supplementary Figure 8. Topography of a Au/PCCO/STO sample. a, **b**, Typical topography of a Au thin film (10 nm) on PCCO/STO (**a**) and corresponding 3d image (**b**). **c**, **d**, Height profiles along the two lines in (**a**) are reported in (**c**) and (**d**) in the same colour as the corresponding line scan.

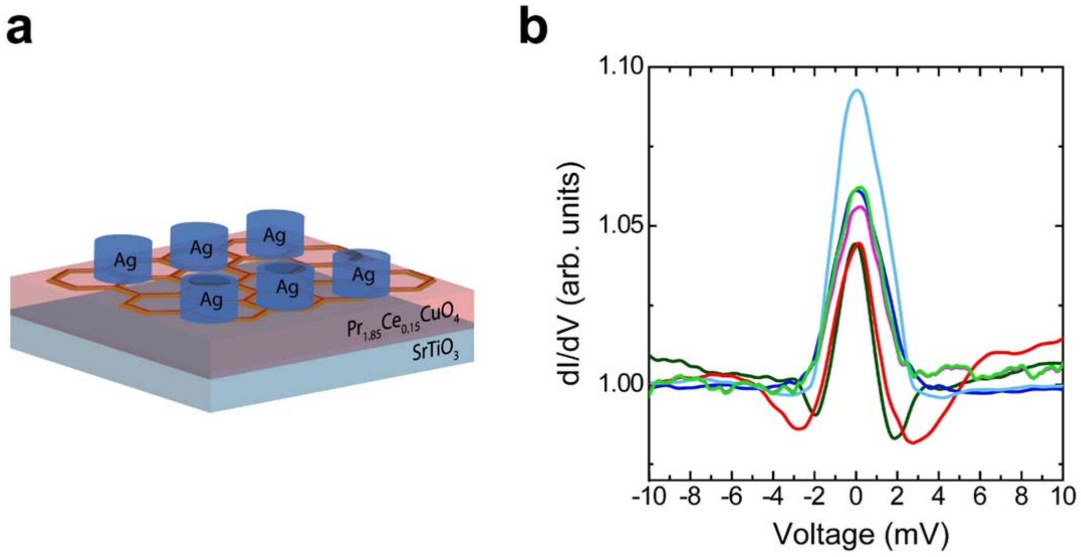

Supplementary Figure 9. Density of states measurements on SLG/PCCO/STO with Ag microislands. (**a**) Schematic of sample structure. (**b**) Scanning tunnelling differential conductance as a function of bias voltage additional to those reported in Fig. 3c of the main text, measured at 4.2 K for a sample with Ag microislands on SLG (hexagonal lattice) on PCCO/STO. The tunneling spectra shown are recorded on the SLG surface near three different Ag microislands. The spectra recorded near the same Ag microisland are labelled in (**b**) with similar colours: blue and light blue for Ag microisland 1, red and pink for Ag microisland 2, green and light green for Ag microisland 3.

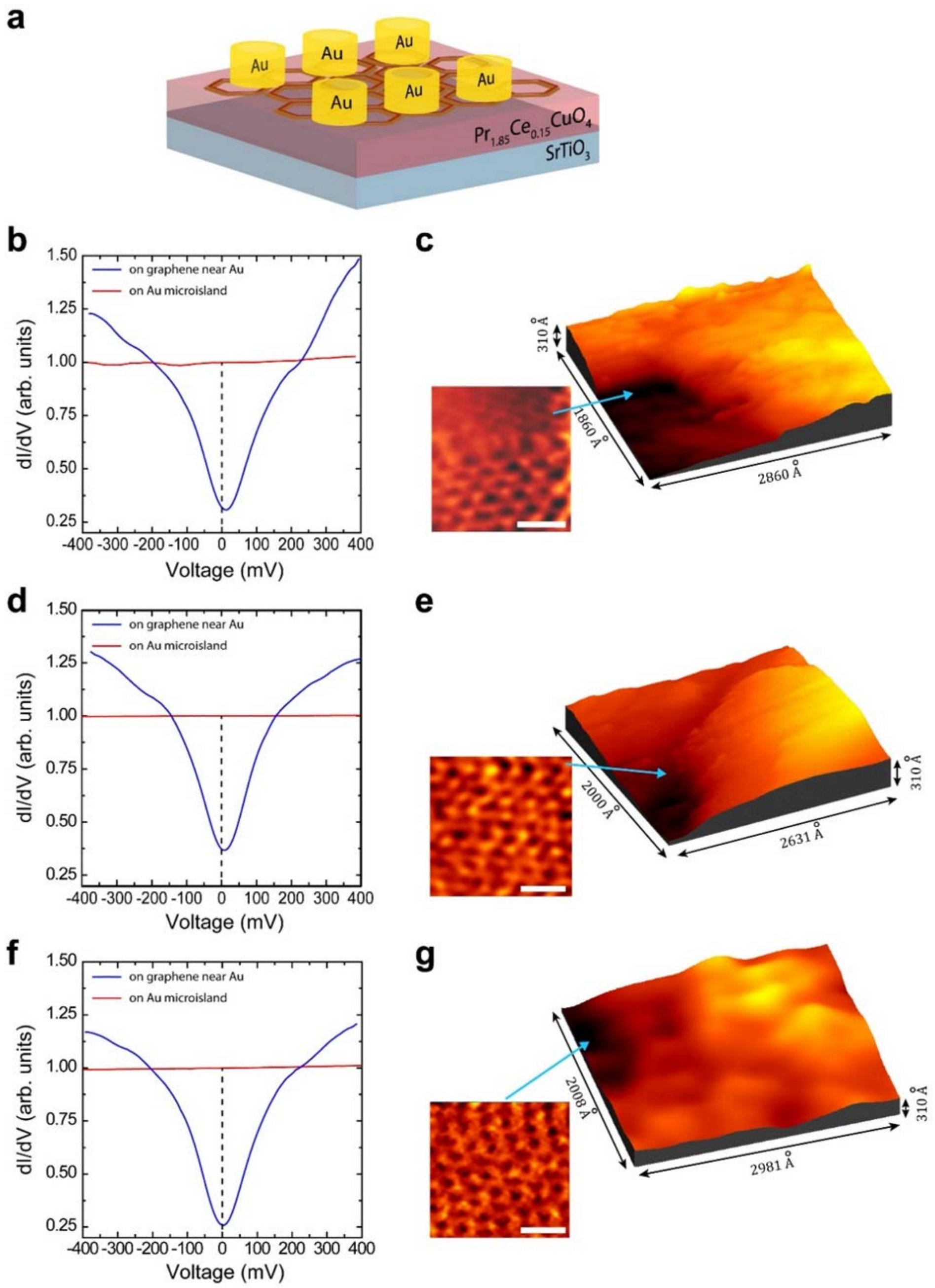

Supplementary Figure 10. Density of states measurements on SLG/PCCO/STO with Au microislands. (**a)** Schematic of sample structure (the hexagonal lattice represents SLG). **b**, **c**, **d**, **e**, **f**, **g**, Scanning tunnelling differential conductance as a function of bias voltage measured at 4.2 K for samples with Au microislands on SLG/PCCO/STO. The tunneling spectra are measured both on SLG near Au microislands (blue curves) and directly on Au microislands (red curves). The tunneling spectra in (**a**), (**d**), (**f**) are measured near the corresponding Au microislands with topography shown in (**c**), (**e**) and (**g**), respectively (the scale bars in **c**, **e**, **f** have a length of 0.5 nm).

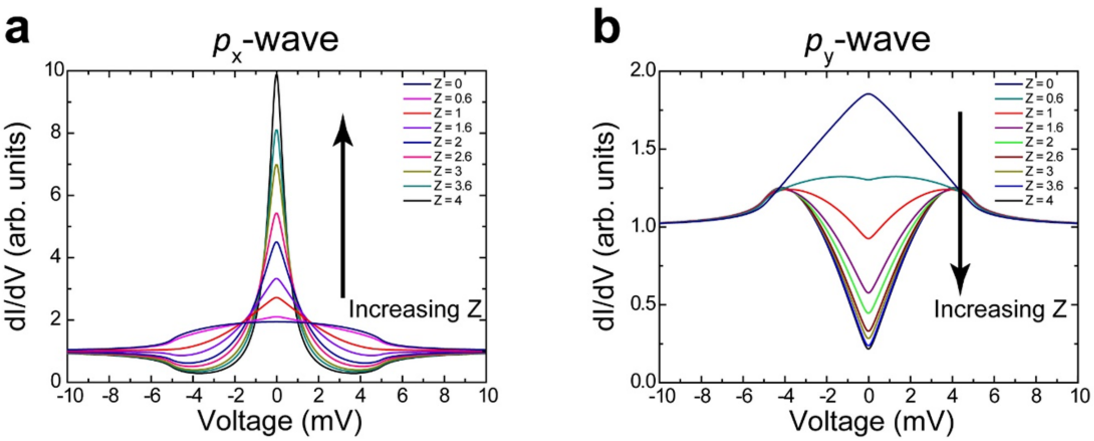

Supplementary Figure 11. Theoretical density of states for SLG on PCCO. **a**, **b**, Theoretical density of states spectra as a function of *Z* for SLG/PCCO under the hypothesis of induced (**a**) p_x-wave and (**b**) p_y-wave symmetry in SLG.

Supplementary References

1. Calizo, I., Balandin, A. A., Bao, W., Miao, F. & Lau, C. N. Temperature dependence of the Raman spectra of graphene and graphene multilayers. *Nano Lett.* **7**, 2645-2649 (2007).
2. Ferrari, A. C. & Robertson, J. Interpretation of Raman spectra of disordered and amorphous carbon. *Phys. Rev. B* **61**, 14095-14107 (2000).
